# Aspartate aminotransferase to platelet ratio index for the assessment of liver fibrosis severity in patients with chronic hepatitis

**Published:** 2011-07-01

**Authors:** Roxana Sirli, Ioan Sporea

**Affiliations:** 1 Department of Gastroenterology, University of Medicine and Pharmacy, Timisoara, Romania; 2 Department of Hepatology, University of Medicine and Pharmacy, Timisoara, Romania

**Keywords:** Aspartate aminotransferase, Liver fibrosis, Chronic hepatitis

Dear Editor,

I read with interest the article by Yilmaz et al. regarding the value of aspartate aminotransferases (AST)-to-platelet ratio index (APRI score) for the noninvasive assessment of liver fibrosis in chronic hepatitis, which was published in Hepatitis Monthly [1]. The authors evaluated patients diagnosed with chronic hepatitis C and B and nonalcoholic fatty liver disease (NAFLD) and assessed their APRI scores to predict the presence of fibrosis (Metavir score of at least F1). Most published studies have used acoustic radiation force impulse (ARFI) elastography results as a predictor of significant fibrosis (Metavir score of F ≥ 2) and cirrhosis in chronic hepatitis C virus (HCV) infections. A meta-analysis [2] from 2007 proved that with a cut-off value of 0.5, APRI results had 81% sensitivity (Se) and 50% specificity (Sp) in predicting significant fibrosis (Metavir score of F ≥ 2) and that with a cut-off value of 1, the Se and Sp for predicting cirrhosis were 76% and 71%, respectively. In a recent meta-analysis that included more than 8,700 patients [3], the summary of areas under receiver operating characteristic (AUROC) values of APRI for the diagnosis of significant fibrosis, severe fibrosis, and cirrhosis were 0.77, 0.80, and 0.83, respectively. For significant fibrosis, the Se and Sp of an APRI threshold of 0.7 were 77% and 72%, respectively, and the corresponding values obtained with a threshold of 1.0 for severe fibrosis were 61% and 64%, respectively. For cirrhosis, the Se and Sp of an APRI threshold of 1.0 were 76% and 72%, respectively [3]. In the study by Yilmaz et al.[1], for an optimal cut-off point of > 0.44, the APRI score was a poor predictor of fibrosis (F ≥ 1), with an Se and Sp of 72.7% and 62.4% (AUROC = 0.582), which was expected since all noninvasive tests show poor performance in differentiation of the early stages of fibrosis.

Regarding hepatitis B virus (HBV) infection, a recently published study from China [4] showed that age could be a factor influencing the ARFI threshold that separates patients without fibrosis from those with a Metavir score of at least F1, the cut-off points being 0.11 for patients aged < 35 years and 0.18 for those >35 years. A study from France showed that the APRI values (0.28 vs. 0.43; P < 0.0001) were significantly lower in inactive hepatitis B surface antigen (HBsAg) carriers than in patients with chronic HBV infection [5]. Although in the study by Yilmaz et al.[1] in patients with chronic HBV infection, the APRI score could not help differentiate subjects with a Metavir score of F0 from those with a Metavir score of at least F1, there are other published data showing that APRI can be a valuable predictor of significant fibrosis (F ≥ 2) and cirrhosis, similar to its predictive value in patients with chronic HCV infection [6], with AUROC values of 0.81 (0.74-0.87) and 0.83 (0.77-0.90), respectively. Few studies have been published regarding the value of APRI in NAFLD, and these studies showed that the APRI values tended to increase with the severity of fibrosis [7][8]. Further studies are required to validate these findings. Overall, considering the wide availability and low cost of performing APRI, we think that it can be a useful tool for the evaluation of fibrosis in patients with chronic hepatitis, possibly in association with other tests, or for repetitive evaluation to assess the progression of fibrosis [9].
